# Quality of life and its related-influencing factors in patients with cervical cancer based on the scale QLICP-CE(V2.0)

**DOI:** 10.1186/s12905-024-03068-1

**Published:** 2024-05-07

**Authors:** Huiyan Chen, Lin Zhou, Daniel Fong, Yingli Cun, Zheng Yang, Chonghua Wan

**Affiliations:** 1 Key Laboratory for Quality of Life and Psychological Assessment and Intervention, The First Dongguan Affiliated Hospital of Guangdong Medical University, Guangdong Medical University, Dongguan, 523808 China; 2 School of Public Health, Guangdong Medical University, Dongguan, 523808 China; 3 School of Nursing, The University of Hong Kong, Hong Kong, China; 4 Yunnan Tumor Hospital, Kunming, 650106 Yunnan Province China

**Keywords:** Cervical cancer, Scale, Health related quality of life, Influencing factors

## Abstract

**Background:**

Quality of life research can guide clinical workers to adopt more targeted treatment and intervention measures, so as to achieve the purpose of improving patients’ quality of life. The objective of this study was to evaluate health-related quality of life in Chinese patients with cervical cancer and to explore its influencing factors.

**Methods:**

A total of 186 patients with cervical cancer were investigated by using the QLICP-CE (V2.0) scale (Quality of Life Instruments for Cancer Patients-Cervical Cancer) developed by our group in China. The data were analyzed by t-test, one-way ANOVA, univariate analysis, and multivariate linear regression.

**Results:**

The total score of quality of life scale for cervical cancer patients was (62.58 ± 12.69), Univariate analysis of objective clinical indexes showed that creatinine concentration was a negative influence factor in the psychological domain, potassium ion concentration was a negative influence factor in the common symptoms and side effect domain, erythrocyte content was a positive influence factor physical domain and common general domain. Multiple linear regression results suggested that clinical staging was the influencing factor of common symptom and side effect domain, common general module and total score of scale. Marital status has different degrees of influence on the psychological, social, and common general domains. The level of education also influenced scores in the social domain.

**Conclusion:**

The total score of quality of life in patients with cervical cancer who received active treatment was acceptable. Marital status, clinical staging, and educational level are the factors that affect the quality of life of patients with cervical cancer. At the same time, potassium ion concentration, red blood cell count and creatinine concentration also have important effects on quality of life in patients with cervical cancer. Therefore, it is very important to give personalized treatment and nursing to patients based on various factors.

## Introduction

Cervical cancer is a common gynecological malignant tumor, with a mortality rate of over 50% [1]. It can be a life-long disease that may seriously threaten women’s health and life [2, 3]. According to the IARC (International Agency for Research on Cancer) reports [4], cervical cancer accounted for 3.1% of all malignant tumors, with 604,127 new cases and 341,831 deaths in 2020. Among female malignant tumors worldwide, cervical cancer ranks the fourth in morbidity and mortality, accounting for 6.5% of new cases and 7.7% of deaths, respectively. Cervical cancer is more prevalent in developing countries. In 2020, there were 110,000 new cases of cervical cancer and 590,00 deaths in China. Cervical cancer ranked sixth in the incidence of female malignant tumors, accounting for 5.2% of all female malignant tumors [5].

With the change of modern disease spectrum and biomedical model, the main goal of tumor treatment has been gradually developed from prolonging survival to improving the overall health status of patients [6]. Health-related quality of life (HRQOL) has been used as an indicator to evaluate the overall health status of patients. HRQOL assessment can be applied not only to guide the selection of clinical protocols and evaluate therapeutic effects, but it may also be used to evaluate prognosis and long-term survival status. Although modern treatment methods have largely reduced pain suffered by patients with cervical cancer, there are still problems that seriously affect the quality of life of patients [7, 8]. Before these problems can be addressed, a proper scale for assessing HRQOL is needed. A large number of cervical cancer specific scales have been developed and applied in clinical practice, for example, FACT-CX 24 [9] (Functional Assessment of Cancer Therapy-Cervix) scale in the United States, and EORTC QLQ-CX24 [10] (European Organization for Research and Treatment of Cancer Quality-of-life Matrimony-Cervical Cancer Module).

Considering culture dependence of QOL, the Chinese QOL instruments system called QLICP (Quality of Life Instruments for Cancer patients) was developed by module approach [11, 12]. The updated second version of the system QLICP V2.0 includes the general module QLICP-GM V2.0 and 22 cancer-specific modules such as breast cancer, bladder cancer, prostate cancer, cervical cancer, leukemia and lymphoma, etc. The QLICP-CE (V2.0) (Quality of Life Instruments for Cancer Patients-Cervical Cancer) scale [13, 14] is a specific scale for patients with cervical cancer among this system. And in this paper, the QLICP-CE (V2.0) scale was used to investigate and evaluate the quality of life of patients with cervical cancer and explore its influencing factors. It is a new attempt to use Chinese native scale to measure HRQOL of Chinese patients with cervical cancer, which provides a reference for further research on health status of cervical cancer patients.

## Methods design

This was a multi-center cross-sectional study on patients with cervical cancer admitted to the Affiliated Hospital of the Guangdong Medical University and the Yunnan Cancer Hospital from April 2016 to mid-June 2017.

### Subjects

186 subjects were included in our study, patients responding to the following criteria were included in our study: (1) Patients adults (≥ 18 years), (2) Patients were diagnosed of cervical cancer, (3) Patients attained primary school education or above to ensure they could self-complete the questionnaire. The exclusion criteria were as follows: (1) Those who had cognitive dysfunction or other mental diseases were excluded, (2) The patients were illiterate and unwilling to participate in the study. Based on previous studies [14], the sample size of this study was about 10–20 times the number of items in the cervical cancer specific scale of the questionnaire, the estimated sample size for this study was 120–240.

An investigator, who was a medical doctor, explained the purpose, content and form-filling requirements of the study to the patient. Consent was sought before a patient would self-complete the study questionnaire.

### Survey instruments

We administered the QLICP-CE V2.0 [14, 15], which comprised a cancer generic module QLICP-GM (V2.0) and a cervical cancer specific module. It covers 5 domains including the physical domain (PHD), psychological domain (PSD), social domain (SOD), Common symptoms and side effect domain (SSD), and specific domain (SPD). There were 44 items, each responded on a 5-point Likert scale from 1 to 5 points. The positively expressed items were assigned a score between 1 and 5, whilst the negatively stated items need to be reversed to calculate the score. Each domain score was derived by aggregating the item scores inside the domain. The total score on the scale was the sum of the domain scores. In order to facilitate comparison, the Raw Score (RS) of each dimension/total score was converted into Standard Score (SS) by using the range method, which was formulated as SS= (RS-Min)×100/R, where Min is the minimum score of this dimension/total score, and the higher the score, the better the quality of life. The QLICP-CE was developed based on the Chinese cultural background, and has been demonstrated with good reliability and validity for assessing the QOL in patients with cervical cancer in China [13, 14] .

Moreover, we also collected data on age, ethnicity, occupation, marital status, educational level, family economic status, treatment methods, clinical staging and clinical classification.

### Statistical analysis

The IBM SPSS 26.0 was used to establish the database and analyzed the data. Descriptive statistics were used to summarize the socio-demographic and clinical indicators, scores in various domains of the QLICP-CE.

Age, ethnicity and marital status were converted into 2 categorical variables and assigned values of 1 and 2 respectively. Educational level and clinical staging were converted into 3 classification variables and assigned values of 1, 2 and 3 respectively (see Table 1 for specific assignment). The 9 demographic sociological indicators and 49 clinical objective indicators collected were simply correlated with scores of the 6 domains and total score of the scale. We analyzed the association of domain scores with socio-demographic and clinical variables, by t-tests and one-way ANOVA. And then LSD-T test was used for pair comparative analysis.

**Table 1:** Classification variable assignment table

variables	The assignment
Age	≤ 35 = 1, >35 = 2
Ethnicity	Han = 1, Others = 2
Marital status	Married = 1, Single (Divorced/separated/widowed) = 2
Educational level	Primary school = 1, Junior high school = 2, Senior high school and above = 3

Multiple stepwise linear regression analyses (backward selection method) were performed to screen the influencing factors with the total score and the score of each domain being as the dependent variables respectively with entry standard ɑin ≤ 0.05, exclusion standard ɑout ≥ 0.10, and test level ɑ=0.05. And marital status, educational level and clinical staging, creatinine concentration, potassium content and red blood cell count, which were statistically significant factors in univariate analysis, were used as independent variables in multiple stepwise linear regression. The categorical independent variables were recoded (assignment) before stepwise linear regression analysis (see Table 1 in detail).

In regard to regression diagnosis on mode assumptions, VIF was used in this study for checking the presence of multi-collinearity among variables in multiple linear regression with the results indicating no multi-collinearity because of all VIF<5. There was no heteroscedasticity with Levene Statistic = 1.760, *P* > 0.05. The DW (Durbin- Watson) statistic was used to test the autocorrelation of the errors with the results showing no autocorrelation (DW = 1.090).

## Results

### Sample characteristics

A total of 186 cervical cancer patients consented to participate in the study. Their average age was 44.6 years (SD = 8.62, range = 24 to 64). Table 2 summarizes the demographics. There were 121 (65.1%) patients only received surgical treatment, 113 (60.8%) were farmers, and 155 (83.3%) were ethnic Han, 150 (80.6%) were married patients, 135 (72.6%) were squamous cell carcinoma, 79 (42.5%) patients and 88 (47.3%) patients were in clinical staging I and II, respectively, 127 (68.28%) patients had junior high school education level or below. Table 3 summarizes scores of the QLICP-CE.

**Table 2:** Socio-demographic and clinical characteristics of the Sample (*n* = 186)

Characteristics	N	%	Characteristics	N	%
**Age**			**Treatments**		
≤ 35	35	18.8%	Surgical Treatment	121	65.1%
>35	151	81.2%	Surgery + Chemotherapy	49	26.3%
**Marital status**			No-surgical treatment	16	8.6%
Married	150	80.6%	**Clinical classification**		
Single (Divorced/separated/widowed)	36	19.4%	Squamous cell carcinomas	135	72.6%
			adenocarcinoma	40	21.5%
**Occupation**			Gland scale cancer	11	5.9%
Worker	25	13.4%			
Farmer	113	60.8%	**Clinical staging**		
Others	48	25.8%	I	79	42.5%
			II	88	47.3%
**Ethnicity**			III	19	10.2%
Han	155	83.3%			
Others	31	16.7%	**Educational level**		
**Family financial status**			Primary school	65	34.9%
Poor Fair High	106 74 6	60.0% 39.7% 0.3%	Junior high school Senior high school and above	62 59	33.3% 31.7%

**Table 3:** Summary of domains of the QLICP-CE V2.0 among 186 patients with cervical cancer

Domains	Minimum	Maximum	Median	Mean	SD
Physical	18.75	93.75	56.25	55.75	15.06
Psychological	19.44	94.44	55.56	55.90	16.76
Social	15.63	96.88	59.38	60.32	14.28
Common symptoms and side	10.71	100.00	66.07	64.90	21.07
Cervical cancer specific	28.00	89.00	58.00	67.07	20.14
Common general	12.50	100.00	68.75	58.90	11.92
Total	35.29	93.63	60.78	62.58	12.69

### Univariable analysis of factors associated with quality of life

Tables 4 and 5 show results of the univariable analysis and simple correlation analysis of factors associated with QOL. With the exception of scores of PHD and SPD, the differences of scores in other dimensions and total scale were statistically significant (P_max_ ≤ 0.02) in different marital status, with married people score being higher in these dimensions than single (Divorced/separated/widowed). The patients who attained high school education or above had significantly higher scores in PHD, SOD, CGD and TOT (P_max_ ≤ 0.02) than those patients with primary school education. Patients with higher education had higher quality of life scores. Those with primary school education scored lower than those with junior high school education, and those with junior high school education scored lower than those with senior high school or above. There were statistically significant differences in PHD, SOD, SSD, CGD and TOT scores among different clinical staging (*P* < 0.05). Patients with clinical staging I had higher scores in the five domains mentioned above than patients with staging II, while patients with staging II had higher scores than patients with staging III. The lower the clinical staging, the higher the scores in each domain and the total quality of life. In addition, a significantly negative correlation was observed between creatinine concentrations and the PSD (*r* = -0.151), and between potassium ion concentration and SSD (*r* = -0.177). In contrast, a RBC count was significantly and positively correlated with PHD (*r* = 0.180) and CGD (*r* = 0.145).

**Table 4:** Univariable analysis of the effects of socio-demographic characteristics on the quality of life of cervical cancer patients

Variables	N	PHD	PSD	SOD	SSD	SPD	CGD	TOT
Age								
≤ 35	35	57.68 ± 15.65	55.79 ± 18.29	57.76 ± 17.05	63.47 ± 22.29	65.77 ± 20.18	58.05 ± 13.70	61.65 ± 14.09
>35	151	55.30 ± 14.93	55.92 ± 16.45	60.91 ± 13.55	65.23 ± 20.84	67.37 ± 20.18	59.09 ± 11.52	62.79 ± 12.39
P		0.40	0.97	0.24	0.66	0.67	0.65	0.63
Ethnicity								
Han	155	54.84 ± 14.64	54.87 ± 16.46	59.44 ± 14.46	64.68 ± 20.98	66.67 ± 20.20	58.34 ± 11.68	61.86 ± 12.50
Others	31	60.28 ± 16.54	61.02 ± 17.59	64.72 ± 12.61	66.01 ± 21.86	69.09 ± 20.01	61.68 ± 12.90	66.18 ± 13.27
P		0.07	0.06	0.06	0.75	0.54	0.16	0.08
Occupation								
Workers	25	56.63 ± 13.18	58.78 ± 19.98	64.38 ± 14.69	64.29 ± 21.78	70.75 ± 17.03	60.20 ± 11.17	65.63 ± 12.33
Famers	113	56.14 ± 15.65	55.21 ± 16.82	58.93 ± 13.15	64.25 ± 22.07	67.35 ± 20.85	58.62 ± 12,66	62.10 ± 12.84
Others	48	54.36 ± 14.75	56.02 ± 14.93	61.46 ± 16.29	66.74 ± 18.45	64.50 ± 19.95	58.88 ± 10.64	62.10 ± 12,55
P		0.76	0.63	0.18	0.78	0.44	0.84	0.44
Marital status								
Married	150	56.71 ± 14.89	57.30 ± 16.74	62.15 ± 14.43	66.83 ± 21.23	67.81 ± 20.05	60.33 ± 12.06	63.65 ± 13.11
Single	36	51.74 ± 15.34	50.08 ± 15.80	52.69 ± 10.80	56.85 ± 18.61	64.00 ± 20.49	52.92 ± 9.31	58.10 ± 9.68
P		0.08	0.02	0.00	0.01	0.31	0.00	0.01
Educational level								
Primary school	65	54.04 ± 14.85	53.85 ± 14.66	57.60 ± 11.56	62.58 ± 21.41	66.09 ± 18.57	56.80 ± 11.05	60.71 ± 10.12
Junior high school	62	53.33 ± 12.56	54.17 ± 14.71	58.72 ± 12,57	64.34 ± 20.72	65.15 ± 20.07	57.53 ± 10.40	60.94 ± 11.22
Senior high school and above	59	60.17 ± 16.87	59.98 ± 20.16	64.99 ± 17.42	68.04 ± 21.04	70.16 ± 21.79	62.64 ± 13.55	66.35 ± 15.71
P		0.02	0.08	0.01	0.35	0.35	0.01	0.02
Family financial status								
Poor	106	56.04 ± 14.95	54.85 ± 16.17	58.31 ± 12.58	64.25 ± 21.74	65.72 ± 20.87	57.81 ± 11.79	61.69 ± 12.35
Fair	74	55.62 ± 15.36	57.58 ± 17.50	62.96 ± 14.95	65.40 ± 20.11	69.57 ± 18.75	60.34 ± 12.02	63.95 ± 12.79
High	6	52.08 ± 15.27	53.70 ± 19.06	63.02 ± 27.49	70.24 ± 23.65	60.07 ± 23.18	60.33 ± 13.23	61.44 ± 17.97
P		0.82	0.53	0.88	0.77	0.31	0.30	0.49
Clinical classification								
Squamous cell carcinomas	135	56.00 ± 14.95	55.35 ± 16.65	60.44 ± 14.39	65.80 ± 20.25	67.58 ± 20.38	59.27 ± 11.38	62.78 ± 12.53
Adenocarcinoma	40	56.09 ± 15.60	57.71 ± 16.56	60.70 ± 14.57	63.39 ± 23.18	66.67 ± 18.79	58.35 ± 13.39	62.49 ± 13.18
Gland scale cancer	11	51.42 ± 15.20	56.06 ± 19.99	57.39 ± 12.60	59.42 ± 23.87	62.31 ± 23.08	56.27 ± 13.59	60.47 ± 13.82
P		0.62	0.74	0.78	0.55	0.70	0.69	0.85
Clinical staging								
I	79	57.75 ± 17.01	58.09 ± 17.69	63.77 ± 14.65	72.33 ± 20.03	69.25 ± 20.85	62.13 ± 12.19	66.02 ± 13.78
II	88	55.65 ± 13.04	54.64 ± 15.80	57.63 ± 14.24	60.19 ± 20.08	66.64 ± 19.83	57.02 ± 11.47	60.63 ± 11.62
III	19	47.86 ± 13.14	52.63 ± 15.80	58.39 ± 9.61	55.83 ± 20.93	59.98 ± 17.59	54.16 ± 9.77	57.28 ± 8.94
P		0.04	0.28	0.02	0.00	0.19	0.00	0.00
Treatment received								
Surgery	121	55.79 ± 15.88	56.45 ± 16.98	60.95 ± 13.82	66.74 ± 21.41	66.31 ± 21.10	59.45 ± 11.93	63.05 ± 13.09
Surgery + Chemotherapy	49	56.70 ± 13.55	55.22 ± 17.23	59.12 ± 16.51	62.90 ± 20.00	70.28 ± 19.50	58.73 ± 12.82	62.71 ± 13.00
Non-surgical treatment	16	52.54 ± 13.37	53.82 ± 14.12	59.18 ± 10.24	57.14 ± 20.62	63.02 ± 12.97	55.90 ± 11.92	58.61 ± 7.48
P		0.63	0.80	0.71	0.17	0.36	0.40	0.44

Physical domain, (PHD); Psychological domain, (PSD); Social domain, (SOD); Common symptoms and side effect domain, (SSD); Specific domain, (SPD); Common general domain, (CGD); Total, (TOT)

**Table 5:** Simple correlation analysis of clinical objective indexes in patients with cervical cancer

Correlations									
		PHD	PSD	SOD	SSD	SPD	TOT	CGD	
A-G	Pearson Correlation	-0.080	-0.124	-0.032	-0.023	-0.085	0.022	-0.050	
	Sig.(2-tailed)	0.278	0.091	0.662	0.756	0.250	0.766	0.497	
ALB	Pearson Correlation	0.003	-0.060	0.050	-0.088	-0.070	0.062	-0.023	
	Sig.(2-tailed)	0.966	0.413	0.496	0.230	0.344	0.397	0.754	
ALP	Pearson Correlation	0.055	0.080	0.011	-0.104	-0.026	0.001	0.000	
	Sig.(2-tailed)	0.460	0.279	0.876	0.158	0.727	0.994	1.000	
ALT	Pearson Correlation	0.073	0.076	-0.028	0.061	0.048	-0.045	0.044	
	Sig.(2-tailed)	0.323	0.302	0.707	0.408	0.515	0.542	0.554	
AST	Pearson Correlation	0.034	0.004	0.005	0.040	0.025	-0.064	0.012	
	Sig.(2-tailed)	0.646	0.960	0.943	0.586	0.732	0.386	0.863	
AST-ALT	Pearson Correlation	-0.047	0.107	0.044	-0.033	-0.047	-0.024	-0.052	
	Sig.(2-tailed)	0.524	0.146	0.548	0.655	0.528	0.750	0.477	
BUN	Pearson Correlation	0.072	0.066	0.082	0.005	0.029	0.074	0.062	
	Sig.(2-tailed)	0.328	0.371	0.265	0.948	0.696	0.313	0.398	
Ca	Pearson Correlation	0.022	-0.044	0.036	-0.093	0.004	-0.078	-0.024	
	Sig.(2-tailed)	0.764	0.553	0.628	0.208	0.961	0.291	0.747	
CL	Pearson Correlation	0.035	-0.072	-0.072	0.023	0.016	-0.006	0.000	
	Sig.(2-tailed)	0.633	0.327	0.331	0.753	0.825	0.940	1.000	
CRE	Pearson Correlation	-0.052	-0.151*	-0.021	-0.037	-0.042	-0.027	-0.082	
	Sig.(2-tailed)	0.483	**0.039**	0.777	0.620	0.565	0.710	0.268	
DB	Pearson Correlation	0.017	-0.094	0.017	0.077	0.038	-0.004	-0.011	
	Sig.(2-tailed)	0.819	0.201	0.823	0.295	0.605	0.958	0.896	
Fe	Pearson Correlation	0.063	0.080	0.058	0.036	0.080	0.073	0.086	
	Sig.(2-tailed)	0.394	0.278	0.431	0.628	0.277	0.322	0.245	
GGT	Pearson Correlation	-0.056	0.038	-0.027	-0.080	-0.0122	-0.041	-0.085	
	Sig.(2-tailed)	0.445	0.602	0.710	0.277	0.097	0.581	0.249	
GP	Pearson Correlation	0.103	0.070	0.016	-0.051	0.017	0.036	0.040	
	Sig.(2-tailed)	0.164	0.340	0.828	0.493	0.820	0.628	0.585	
GLU	Pearson Correlation	0.104	0.103	0.067	0.080	0.024	0.100	0.071	
	Sig.(2-tailed)	0.159	0.160	0.365	0.277	0.749	0.173	0.337	
K	Pearson Correlation	-0.058	-0.113	0.037	-0.177*	-0.142	-0.115	-0.108	
	Sig.(2-tailed)	0.428	0.125	0.617	**0.016**	0.053	0.119	0.142	
LDH	Pearson Correlation	-0.023	0.024	0.106	0.056	-0.110	0.048	-0.017	
	Sig.(2-tailed)	0.756	0.744	0.150	0.448	0.135	0.515	0.814	
Mg	Pearson Correlation	0.041	0.019	-0.040	-0.087	0.072	-0.035	0.034	
	Sig.(2-tailed)	0.581	0.796	0.584	0.240	0.330	0.637	0.645	
Na	Pearson Correlation	-0.050	-0.024	0.027	0.081	-0.092	0.030	-0.052	
	Sig.(2-tailed)	0.501	0.749	0.715	0.273	0.210	0.686	0.479	
P	Pearson Correlation	0.048	0.019	-0.037	-0.091	0.086	-0.035	0.043	
	Sig.(2-tailed)	0.517	0.793	0.621	0.216	0.245	0.631	0.562	
TPA	Pearson Correlation	0.047	0.045	-0.058	0.024	-0.072	0.011	-0.019	
	Sig.(2-tailed)	0.523	0.544	0.4300	0.750	0.327	0.882	0.797	
TBA	Pearson Correlation	-0.013	0.076	0.008	0.018	-0.049	0.025	0.009	
	Sig.(2-tailed)	0.861	0.300	0.915	0.807	0.503	0.739	0.904	
TP	Pearson Correlation	0.012	0.001	0.073	0.057	-0.093	0.048	-0.037	
	Sig.(2-tailed)	0.875	0.994	0.323	0.439	0.205	0.515	0.618	
UA	Pearson Correlation	-0.072	-0.061	-0.047	-0.003	-0.122	-0.053	-0.095	
	Sig.(2-tailed)	0.326	0.407	0.528	0.970	0.096	0.475	0.199	
BASO	Pearson Correlation	0.086	0.023	-0.137	-0.012	-0.029	-0.032	-0.030	
	Sig.(2-tailed)	0.246	0.752	0.063	0.866	0.698	0.669	0.679	
BASO Ratio	Pearson Correlation	0.131	0.046	-0.102	-0.056	0.000	-0.035	-0.008	
	Sig.(2-tailed)	0.075	0.537	0.166	0.450	0.999	0.639	0.913	
EO	Pearson Correlation	0.073	0.061	-0.015	0.018	-0.032	0.019	0.004	
	Sig.(2-tailed)	0.324	0.408	0.841	0.811	0.662	0.792	0.952	
EO Ratio	Pearson Correlation	0.072	0.043	0.008	-0.026	-0.007	-0.011	0.003	
	Sig.(2-tailed)	0.326	0.565	0.911	0.728	0.919	0.882	0.963	
HCT	Pearson Correlation	0.057	-0.014	-0.044	0.036	-0.020	0.018	-0.002	
	Sig.(2-tailed)	0.443	0.851	0.549	0.622	0.786	0.812	0.981	
HGB	Pearson Correlation	0.074	-0.017	-0.035	0.038	-0.019	0.025	0.004	
	Sig.(2-tailed)	0.315	0.816	0.632	0.602	0.795	0.736	0.956	
LYMPH	Pearson Correlation	0.095	0.076	0.016	0.075	-0.030	0.066	0.050	
	Sig.(2-tailed)	0.195	0.302	0.831	0.308	0.680	0.367	0.498	
LYMPH Ration	Pearson Correlation	0.107	0.038	0.069	0.012	0.045	0.039	0.074	
	Sig.(2-tailed)	0.146	0.605	0.353	0.873	0.541	0.599	0.313	
MCH	Pearson Correlation	0.133	0.061	0.041	0.081	0.014	0.094	0.080	
	Sig.(2-tailed)	0.070	0.411	0.574	0.271	0.854	0.201	0.279	
MCHC	Pearson Correlation	0.045	0.042	0.137	0.085	-0.015	0.119	0.061	
	Sig.(2-tailed)	0.540	0.572	0.062	0.248	0.837	0.106	0.407	
MCV	Pearson Correlation	0.126	0.103	0.057	0.104	0.024	0.110	0.101	
	Sig.(2-tailed)	0.086	0.163	0.440	0.159	0.742	0.136	0.169	
MONO	Pearson Correlation	-0.113	-0.028	0.022	-0.054	-0.076	-0.082	-0.070	
	Sig.(2-tailed)	0.125	0.701	0.765	0.468	0.302	0.267	0.339	
MONO Ratio	Pearson Correlation	0.042	-0.024	-0.012	-0.104	0.008	-0.072	-0.041	
	Sig.(2-tailed)	0.567	0.750	0.874	0.158	0.918	0.332	0.577	
NEUT	Pearson Correlation	-0.143	-0.067	-0.141	-0.004	-0.013	-0.067	-0.085	
	Sig.(2-tailed)	0.052	0.366	0.055	0.961	0.863	0.361	0.249	
PLT	Pearson Correlation	-0.016	-0.047	-0.044	-0.075	-0.017	-0.102	-0.046	
	Sig.(2-tailed)	0.831	0.528	0.550	0.307	0.819	0.167	0.533	
RBC	Pearson Correlation	0.180*	0.057	0.028	0.118	0.113	0.145*	0.114	
	Sig.(2-tailed)	**0.014**	0.436	0.708	0.108	0.125	**0.048**	0.123	
RDW	Pearson Correlation	0.034	0.039	-0.010	0.015	-0.022	0.027	0.008	
	Sig.(2-tailed)	0.644	0.599	0.890	0.839	0.769	0.717	0.915	
WBC	Pearson Correlation	-0.099	0.035	-0.028	0.000	-0.092	-0.025	-0.053	
	Sig.(2-tailed)	0.181	0.633	0.706	0.997	0.211	0.737	0.469	
APTT	Pearson Correlation	0.056	0.013	0.056	0.038	0.002	0.050	0.038	
	Sig.(2-tailed)	0.449	0.859	0.451	0.610	0.976	0.496	0.605	
Fg	Pearson Correlation	-0.053	0.089	0.017	-0.008	-0.010	-0.011	0.012	
	Sig.(2-tailed)	0.473	0.225	0.815	0.916	0.891	0.883	0.873	
PTRATIO	Pearson Correlation	-0.096	-0.010	0.011	-0.039	-0.040	-0.031	-0.051	
	Sig.(2-tailed)	0.193	0.890	0.885	0.596	0.588	0.674	0.492	
PT	Pearson Correlation	-0.086	-0.024	0.019	-0.025	-0.026	-0.020	-0.045	
	Sig.(2-tailed)	0.244	0.749	0.801	0.736	0.722	0.789	0.543	
TT	Pearson Correlation	0.080	0.082	-0.089	0.025	-0.071	0.028	-0.001	
	Sig.(2-tailed)	0.275	0.263	0.229	0.736	0.335	0.702	0.990	
CA125	Pearson Correlation	-0.082	0.054	0.054	0.077	0.089	0.031	0.055	
	Sig.(2-tailed)	0.269	0.463	0.466	0.293	0.227	0.671	0.457	
CA199	Pearson Correlation	0.085	0.025	-0.031	-0.025	0.115	0.022	0.076	
	Sig.(2-tailed)	0.250	0.731	0.678	0.735	0.117	0.763	0.303	

* Correlation is significant at the 0.05 level (2-tailed). A-G, Albumin globulin ratio; ALB, albumin; ALP, Alkaline phosphatase; ALT, Alanine aminotransferase; AST, Aspartate aminotransferase; BUN, The urea; Ca, calcium; CL, chlorine; CRE, creatinine; DB, Direct bilirubin; GGT, Rglutamine transpeptidase; GP, globulin; GLU, glucose; LDH, Lactate dehydrogenase; TPA, Before total protein; TBA, Total bile acid; TP, The total protein; UA, Uric acid; BASO, basophil; EO, eosinophils; HCT, hematocrit; HGB, hemoglobin; LYMPH, Absolute value of lymphocytes; MCH, Mean hemoglobin; MCHC, Mean hemoglobin concentration; MCV, Mean erythrocyte volume; MONO, Monocyte absolute value; NEUT, Absolute value of neutrophil; PLT, platelet; RBC, Red blood cells; RDW, Red cell distribution width; WBC, White blood cells; APTT, Activated partial prothrombin; Fg, Plasma fibrinogen; PTRATIO, Prothrombin time ratio; PT, Prothrombin time; TT, Plasma thrombin time; CA125,Carbohydrate antigen125;CA199,Carbohydrate antigen199

### Multiple linear regression on influencing factors of quality of life

Table 6 shows the results of multiple linear regression on influencing factors of quality of life. It can be seen that patients with higher PHD were those who attained a higher educational level, or a higher RBC. Patients with higher PSD were those who were married, or a lower CRE. Patients with higher SOD were those who were married, or a higher educational level. Patients with higher SSD were those who were in a lower clinical staging of the disease, or a lower potassium ion concentration. Patients with higher CGD were those who were married, or a lower clinical staging of the disease. Furthermore, patients with higher TOT were those who were in a lower clinical staging of the disease.

**Table 6:** Multiple linear regression analysis of factors associated with quality of life in patients with cervical cancer

Domains	Factors	B	Std. Error	Standardized B	P
PHD^a^	Educational level	3.28	1.32	0.18	0.01
	RBC	0.92	0.34	0.19	0.00
PSD^b^	Marital status	-7.65	3.05	-0.18	0.01
	CRE	-0.23	0.10	-0.16	0.03
SOD^c^	Marital status	-8.62	2.55	-0.24	0.00
	Educational level	3.11	1.23	0.18	0.01
SSD^d^	Clinical staging	-9.83	2.24	-0.30	0.00
	K	-0.08	0.03	-0.19	0.01
CGD^e^	Marital status	-5.75	2.23	-0.19	0.01
	Clinical staging	-3.32	1.35	-0.18	0.01
TOT^f^	Clinical staging	-4.74	1.39	-0.24	0.00

Physical domain, (PHD); Psychological domain, (PSD); Social domain, (SOD); Common symptoms and side effect domain, (SSD); Specific domain, (SPD); Common general domain, (CGD); Total, (TOT); B,β-coefficient; PHD^a^:*P*<0.01,Adjusted-R^2^ = 0.054;PSD^b^:*P*<0.01,Adjusted-R^2^ = 0.045;SOD^c^:*P*<0.001,Adjusted-R^2^ = 0.09;SSD^d^:*P*<0.001,Adjusted-R^2^ = 0.114;CGD^e^:*P*<0.001,Adjusted-R^2^ = 0.081;TOT^f^:*P*<0.01,Adjusted-R^2^ = 0.054

## Discussions

### Main findings of the study

The present study evaluated the Quality of Life (QOL) in patients with cervical cancer, and the association between socio-demographic variables, clinical objective indicators and QOL in the Chinese population.

The total score of the QLICP-CE scale for cervical cancer patients was (62.58 ± 12.69). Univariate analysis of objective clinical indexes showed that creatinine concentration was a negative influence factor in the psychological domain, potassium ion concentration was a negative influence factor in the common symptoms and side effect domain, erythrocyte content was a positive influence factor physical domain and common/general domain. Multiple linear regression results displayed that clinical staging was the influencing factor of common symptom and side effect domain, common/general module and total score of scale. Marital status has different degrees of influence on the psychological, social, and common/ general domains. The level of education also influenced scores in the social domain.

### Implications and comparison with Literatures

In the process of reading a large number of literatures, we learned that age, occupation, ethnicity and pathological types were regularly distributed in patients with cervical cancer, and our findings also showed this phenomenon. The age at which cervical cancer occurs varied from country to country. Early foreign data showed that the incidence of cervical cancer in women aged 25–35 years had a significant upward trend. In China, cervical cancer was mostly seen after the age of 35, and the most frequent age was between 45 and 50 [16, 17]. Among the 186 patients with cervical cancer in this survey, 81.2% (151/186) were > 35 years old, which was consistent with the situation of the age of cervical cancer in China reported previously. In terms of occupation, most of the patients were farmers, which was mainly related to their low income and limited access to health information. In terms of ethnicity, Han nationality was the most affected in this study, followed by other minority groups, which could be related to the fact that most of the local permanent residents were Han. In terms of pathological types of cervical cancer, there were also differences among different countries and nationalities, but the differences were not significant [18, 19]. Among the subjects of this survey, squamous cell carcinoma accounted for a large proportion (72.6%), which was consistent with the fact that squamous cell carcinoma was also the most common cancer in China [20, 21].

According to the scores of cervical cancer patients in various domains, the decreasing order is SPD, SSD, TOT, SOD, CGD, PSD and PHD. PHD reflects the physical function of patients. In addition to the impact of cervical cancer itself on patients, the adverse reactions left by radiotherapy and chemotherapy may have a significant impact on the patient’s body, and affect the long-term quality of life of patients, so the score of PHD is not high. PSD reflects the psychological function of patients. The average age of patients in this study is (44.62 ± 8.62) years old, whose menopausal symptoms and sexual dysfunction will lead to fatigue, anxiety and even depression [22, 23], and cervical cancer patients have lower scores in PSD compared with other cancer patients [24].

Creatinine concentration was a negative factor of mental function. Creatinine is one type of muscles that produces toxins, mainly by renal clearance. A high concentration of creatinine in the body may cause a series of electrolyte metabolism disorder or even multiple system function disorder, resulting in the manifestation of clinical symptoms that increases the patient’s mental load, and poor quality of life [25]. RBC count was a positive factor for PHD. High red blood cell count is beneficial to supply enough oxygen to the tissues and organs, the higher the red blood cell count, the stronger the blood oxygen carrying capacity, and the higher the PHD score, the better the patient’s quality of life. Increase in serum potassium level was associated with reduced scores in the SSD. Potassium ion is the main caution to maintain the physiological activities of cells [26]. When tumor occurs, abnormally increased potassium ion concentration desaturates the osmotic pressure and acid-base balance of cells in the body, leading to a series of metabolic disorders in the body [27], and in a variety of treatment side effects and poor quality of life.

In this study, marital status was dichotomized by married and single (divorced/separated/widowed). Multiple linear regression results indicated that the factor of marital status was included in the regression model with PSD, SOD and CGD as dependent variables respectively. And marriage was a protective factor of quality of life in these three domains, which was consistent with some published research results and reported in domestic and foreign literature [28–31]. Patients that are married can get more family support during the treatment process, with more satisfaction in emotional comfort and financial support. Cancer patients undergo a long treatment cycle, during which may suffer from physical and mental pain. As the disease progresses, patients who are single (divorced/separated/widowed) are more likely to suffer from psychological problems such as depression, anxiety and loneliness, which will affect the quality of life [32, 33].

After stepwise multiple linear regression, in the PHD and SOD domains, educational level was included in the regression model, affecting the social function of patients with cervical cancer, the higher the educational level, the higher the score, the better the quality of life; Studies have shown that patients with higher educational level have more active thinking in dealing with affairs, more ways to find spiritual support, more harmonious ways to communicate with friends and family, and can accept and deal with social interpersonal communication with a more open perspective and mentality [34]. All of these are conducive to cancer patients to relieve the negative psychology caused by illness and depression and lack of social role.

The results of this study suggested that CGD and TOT scores of patients in stage I were higher than those in stage II, while the scores of patients in stage II were higher than those in stage III, this result was consistent with the existing research conclusions. Zhao [35] showed that the quality of life of patients with intermediate and advanced cervical cancer, who was treated for six months, was still lower than that of patients with early cervical cancer. Further multivariate analysis showed that clinical staging of cervical cancer was a factor influencing the total score of multiple domains and scales. It has an important influence on the quality of life of patients with cervical cancer, and the patients with late cervical cancer stage have a poor quality of life. However, some studies suggest that stage has little influence on quality of life in patients with cervical cancer [36]. Cervical cancer patients are clinically characterized by irregular vaginal bleeding and abnormal leucorrhea. Untreated, these symptoms can worsen as the cancer progresses to an advanced stage. Advanced patients are often accompanied by metastasis and cachexia, and the patient’s physique will be significantly reduced compared to the early stage. All these will bring unpleasant physical and mental experience to patients, and they worry that the progress of the disease will endanger life, thus affecting the quality of life. The scale used in this study was self-completed. Thus, illiterate patients who did not attain primary school education were excluded.

### Strengths and limitations of the study

This study was based on the quality of life scale for cervical cancer patients (QLICP-CE V2.0) and explored the indicative roles of general demographic factors and some clinical indicators on their quality of life. The QLICP-CE(V2.0) was developed by modular approach with combination of the general module QLICD-GM and a specific module for cervical cancer. Contrast to other QOL instruments, the QLICP-CE(V2.0) has several advantages [13, 14]. First, it can compare HRQOL across diseases by the general module and also capture the symptoms and side effects by the specific module, demonstrating both generic and specific properties. Second, it consists of a moderate number of items with a clear hierarchical structure (items→ facets→ domains→ overall) so that mean scores can be computed not only at the domain and the overall levels but also at the different facet levels to detect changes in greater detail. Therefore, it could better reflect the quality of life in patients with cervical cancer and is sensitive to the affecting factors.

There are some limitations to this study. First, the patients were sampled only in Guangdong and Yunnan provinces, and the sample may not be fully representative of the general population of Chinese breast cancer patients. Second, there may be other potential factors that affect the QOL of breast cancer patients, which were not comprehensively explored in this study. Besides, only statistically significant variables in the single factor analysis were included in the multiple linear regression model considering there were too many independent variables relative to the sample size and the selection of too many variables would have resulted in a decrease in precision due to the excessive amount of calculations. And thus it could have resulted in omission of some variables with a large interaction and a small individual effect. Last but not least, this study focused on an initial exploration of the demographic and clinical characteristics of factors that may influence the QOL of breast cancer patients, the mechanisms of QOL influencing factors remain unclear especially clinical biochemical indexes, which still need to be further studied.

### Suggestions for further research

On the basis of this study, the multi-center large-sample survey will continue to be conducted to explore more factors. Future studies should investigate why quality of life is linked to socio-demographic and clinical factors, and also explore their mechanisms. In future studies, we will try our best to take objective factors and subjective factors (such as personality characteristics, psychological resilience, etc.) into account to explore the factors affecting the quality of life of patients with cervical cancer. It will provide theoretical support for improving the quality of life of patients with cervical cancer.

## Conclusions

In this study, the total score of the scale was moderate, suggesting that the overall quality of life of patients with cervical cancer who received active treatment after the disease was fairly good. Both the disease and its treatment cause prolonged and inevitable physical pain, patients had the lowest quality-of-life scores in the PHD. The quality of life is related to many factors, not only with the characteristics of demographic sociology, but also with some objective clinical indicators. Marital status, clinical staging, and educational level are the factors that affect the quality of life of patients with cervical cancer. The study also found that potassium ion concentration, red blood cell count and creatinine concentration also had important effects on quality of life in patients with cervical cancer, which are biochemical indicators that have not been reported before. However, in this study, external factors such as clinical staging and education level were mainly considered, while the influence of internal factors such as personality and hobbies on patients’ quality of life was not considered. The results of this study can set the ground work to adopt more targeted individual treatment and intervention measures, so as to improve the quality of life of patients with cervical cancer.

## Data Availability

The datasets generated and/or analysed during the current study are not publicly available due to confidentiality and privacy, but are available from the corresponding author on reasonable request.

## Abbreviations

QLICP-GM: Quality of Life Instruments for Cancer Patients-General Module
QLICP-CE: Quality of Life Instruments for Cancer Patients-Cervical Cancer
TOT: Total
PHD: Physical domain
PSD: Psychological domain
SOD: Social domain
SSD: Common symptoms and side effect domain
SPD: Specific domain
CGD: Common general domain
FACT-Cx: Functional Assessment of Cancer Therapy-Cervix
EORTC QLQ-Cx: European Organization for Research and Treatment of Cancer Quality-of-Life Questionnaire-Cervical Cancer Module
